# Supplementary material for: Mechanism of completion of peptidyltransferase centre assembly in eukaryotes
Source: eLife. 2019 May 22;8:e44904. doi: 10.7554/eLife.44904 (PMC6579518; doi:10.7554/eLife.44904)
Supplement: Supplementary file 1. [file elife-44904-supp1.docx]

**Supplementary Files**

Supplementary file 1A. Data collection, model refinement and validation

|  | **State I** | **State II** | **State III** | **State IV** | **State V** | **State VI** |
| --- | --- | --- | --- | --- | --- | --- |
| **Data collection** |  |  |  |  |  |  |
| Particles | 216,403 | 32,152 | 260,853 | 48,239 | 52,800 | 46,734 |
| Pixel size (Å) | 1.065 | 1.065 | 1.065 | 1.065 | 1.065 | 1.065 |
| Defocus range (μm) | -0.8--3.2 | -0.8--3.2 | -0.8--3.2 | -0.8--3.2 | -0.8--3.2 | -0.8--3.2 |
| Voltage (kV) | 300 | 300 | 300 | 300 | 300 | 300 |
| Electron dose (e^-^Å^2^) | 63 | 63 | 63 | 63 | 63 | 63 |
| **Model composition** |  |  |  |  |  |  |
| Chains | 48 | 47 | 47 | 48 | 47 | 45 |
| Atoms | 134,475 | 129,260 | 128,685 | 130,375 | 129,205 | 124,723 |
| Protein residues | 7,602 | 6,934 | 6,858 | 7,066 | 7,063 | 6,450 |
| RNA bases | 3,423 | 3,423 | 3,423 | 3,423 | 3,424 | 3,441 |
| **Refinement** |  |  |  |  |  |  |
| Resolution range (Å) | 6.3-2.3 | 6.3-2.3 | 6.3-2.3 | 6.3-2.3 | 6.3-2.3 | 6.3-2.3 |
| B-factors (mean) |  |  |  |  |  |  |
| Protein | 53.50 | 58.90 | 62.14 | 77.97 | 67.68 | 56.66 |
| RNA | 57.60 | 78.10 | 69.05 | 76.76 | 70.79 | 67.35 |
| **R.m.s. deviations** |  |  |  |  |  |  |
| Bond lengths (Å) | 0.007 | 0.008 | 0.013 | 0.005 | 0.015 | 0.020 |
| Bond angles (^o^) | 0.873 | 1.004 | 0.965 | 0.846 | 1.174 | 1.481 |
| **Model validation** |  |  |  |  |  |  |
| MolProbity score  (percentile, 0 -99 Å) | 1.60 (92) | 1.99 (76) | 1.60 (92) | 1.65 (91) | 1.73 (88) | 1.86 (83) |
| Clash score, all atoms  (%, all resolutions) | 3.99 (96) | 6.90 (87) | 3.12 (98) | 4.01 (96) | 4.04 (96) | 4.96 (94) |
|  |  |  |  |  |  |  |
|  |  |  |  |  |  |  |
|  |  |  |  |  |  |  |
|  | **State I** | **State II** | **State III** | **State IV** | **State V** | **State VI** |
| **Ramachandran plot** |  |  |  |  |  |  |
| Favoured (%) | 93.73 | 90.54 | 91.62 | 92.53 | 90.43 | 88.69 |
| Allowed (%) | 6.19 | 9.36 | 8.29 | 7.31 | 9.40 | 11.03 |
| Outliers (%) | 0.08 | 0.10 | 0.09 | 0.16 | 0.17 | 0.28 |
| Poor rotamers (%) | 0.87 | 1.27 | 0.40 | 0.25 | 0.64 | 1.04 |
| CaBLAM outliers (%) | 3.18 | 4.07 | 3.57 | 3.87 | 3.94 | 4.17 |
| **Model vs. Map validation** |  |  |  |  |  |  |
| CC_mask_ | 0.88 | 0.82 | 0.85 | 0.82 | 0.85 | 0.84 |
| Resolution masked (0/0.143/0.5) | 3.0/3.2/3.2 | 3.6/3.8/3.9 | 3.0/3.2/3.2 | 3.3/3.5/3.6 | 3.2/3.3/3.3 | 3.1/3.3/3.4 |
| Resolution unmasked (0/0.143/0.5) | 3.1/3.2/3.2 | 3.8/3.9/3.9 | 3.1/3.2/3.2 | 3.5/3.5/3.6 | 3.2/3.3/3.3 | 3.3/3.4/3.4 |
| **RNA** |  |  |  |  |  |  |
| Correct sugar packers (%) | 99.50 | 99.44 | 99.33 | 99.53 | 99.47 | 99.24 |
| Good backbone conformations (%) | 78.97 | 74.53 | 79.05 | 78.59 | 78.18 | 75.21 |

Supplementary file 1B. Summary of modelled ribosomal proteins, assembly factors and rRNA

| **Chain ID** | **Components** | **Length** | **Residues built (State-I)** | **Residues built (State-II)** | **Residues built (State-III)** | **Residues built (State-IV)** | **Residues built (State-V)** | **Residues built (State-VI)** |
| --- | --- | --- | --- | --- | --- | --- | --- | --- |
| A | 25S | 3396 | 3-440, 494-1955, 2094-2530, 2547-3223, 3265-3396 | 3-440, 494-1955, 2094-2530, 2547-3223, 3265-3396 | 3-440, 494-1955, 2094-2530, 2547-3223, 3265-3396 | 3-440, 494-1955, 2094-2530, 2547-3223, 3265-3396 | 3-440, 494-1955, 2094-2530, 2546-3223, 3265-3396 | 3-440, 494-1955, 2094-2530, 2546-3223, 3264-3396 |
| B | uL2 | 254 | 2-248 | 2-248 | 2-248 | 2-248 | 2-248 | 2-248 |
| C | uL3 | 387 | 2-381 | 2-382 | 2-382 | 2-382 | 2-382 | 2-382 |
| D | uL4 | 362 | 2-361 | 2-362 | 2-362 | 2-362 | 2-362 | 2-362 |
| E | uL5 | 174 | 6-174 | 6-174 | 6-174 | 6-174 | 6-174 | 6-174 |
| F | uL6 | 191 | 1-189 | 1-189 | 1-189 | 1-189 | 1-189 | 1-189 |
| G | eL6 | 176 | 2-176 | 2-176 | 2-176 | 2-176 | 2-176 | 2-176 |
| H | eL8 | 256 | 25-247 | 25-247 | 25-247 | 25-247 | 25-247 | 25-247 |
| J | uL13 | 199 | 3-199 | 3-199 | 3-199 | 3-199 | 3-199 | 3-199 |
| K | eL13 | 199 | 2-187 | 2-187 | 2-187 | 2-187 | 2-187 | 2-187 |
| L | uL14 | 137 | 2-137 | 2-137 | 2-137 | 2-137 | 2-137 | 2-137 |
| M | eL14 | 138 | 2-136 | 2-136 | 2-136 | 2-136 | 2-136 | 2-138 |
| N | uL15 | 149 | 2-149 | 2-149 | 2-149 | 2-149 | 2-149 | 2-149 |
| O | eL15 | 204 | 2-204 | 2-204 | 2-204 | 2-204 | 2-204 | 2-204 |
| P | uL18 | 297 | 8-276 | 8-276 | 8-276 | 8-276 | 8-276 | 8-293 |
| Q | eL18 | 186 | 2-186 | 2-186 | 2-186 | 2-186 | 2-186 | 2-186 |
| R | eL19 | 189 | 2-151 | 2-151 | 2-151 | 2-151 | 2-151 | 2-151 |
| S | eL20 | 172 | 2-172 | 2-172 | 2-172 | 2-172 | 2-172 | 1-172 |
| T | eL21 | 160 | 2-160 | 2-160 | 2-160 | 2-160 | 2-160 | 2-160 |
| U | uL22 | 184 | 2-155 | 2-155 | 2-155 | 2-155 | 2-155 | 2-155 |
| V | eL22 | 121 | 11-109 | 11-109 | 11-109 | 11-109 | 11-109 | 11-109 |
| W | uL23 | 142 | 23-142 | 23-142 | 23-142 | 23-142 | 23-142 | 23-142 |
| X | uL24 | 127 | 2-126 | 2-126 | 2-126 | 2-126 | 2-126 | 2-126 |
| Y | eL27 | 136 | 2-136 | 2-136 | 2-136 | 2-136 | 2-136 | 2-136 |
| Z | uL29 | 120 | 3-120 | 3-120 | 3-120 | 3-120 | 3-120 | 3-120 |
| a | eL29 | 59 | 6-57 | 6-57 | 6-57 | 6-57 | 6-57 | 4-57 |
| b | uL30 | 244 | 26-244 | 26-244 | 26-244 | 26-244 | 26-244 | 26-244 |
| c | eL30 | 105 | 9-105 | 9-105 | 9-105 | 9-105 | 9-105 | 9-105 |
| d | eL31 | 113 | 6-112 | 6-112 | 6-112 | 6-112 | 6-112 | 6-112 |
| e | eL32 | 130 | 1-127 | 1-127 | 1-127 | 1-127 | 1-127 | 7-127 |
| f | eL33 | 107 | 2-107 | 2-107 | 2-107 | 2-107 | 2-107 | 2-107 |
| g | eL34 | 121 | 2-104 | 2-104 | 2-104 | 2-104 | 2-104 | 2-104 |
| h | eL36 | 100 | 3-100 | 3-100 | 3-100 | 3-100 | 3-100 | 3-100 |
| I | eL37 | 88 | 2-85 | 2-85 | 2-85 | 2-85 | 2-85 | 2-85 |
| j | eL38 | 78 | 2-78 | 2-78 | 2-78 | 2-78 | 2-78 | 2-78 |
| k | eL39 | 51 | 2-51 | 2-51 | 2-51 | 2-51 | 2-51 | 2-51 |
| l | eL42 | 106 | 2-95 | 2-95 | 2-95 | 2-95 | 2-95 | 2-95 |
| m | eL43 | 92 | 2-90 | 2-90 | 2-90 | 2-90 | 2-90 | 2-92 |
| n | eIF6 | 245 | 1-224 | 1-224 | 1-224 | 1-224 | 1-224 | 1-224 |
| o | Lsg1 | 640 | 133-265, 301-311, 337-512 | 133-267, 301-311, 337-512 | 133-267, 301-311, 337-512 | 133-270, 301-311, 337-512 | 133-264, 301-311, 337-512 | - |
| p | uL1 | 217 | 1-210 (Backbone) | 1-210 (Backbone) | 1-210 (Backbone) | 1-210 (Backbone) | 1-210 (Backbone) | - |
| q | uL16 | 221 | - | - | - | 2-102, 115-217 | 2-217 | 2-217 |
| r | Arx1 | 593 | 19-279, 302-454, 473-562, 567-578 | - | - | - | - | - |
| s | Yvh1 | 364 | 225-352 | 225-352 | - | - | - | - |
| t | eL40 | 128 | - | - | 77-128 | 77-128 | 77-128 | 77-128 |
| u | Rei1 | 393 | 145-261, 300-393 | - | - | - | - | - |
| v | Rpl24 | 155 | 1-60 | 1-60 | 1-60 | 1-60 | 1-60 | 1-60 |
| w | Nmd3 | 518 | 16-404 | 16-404 | 16-404 | 16-404 | 16-147, 156-227, 241-403 | - |
| x | 5S | 121 | 1-121 | 1-121 | 1-121 | 1-121 | 1-121 | 1-121 |
| y | 5.8S | 158 | 1-156 | 1-156 | 1-156 | 1-156 | 1-156 | 1-156 |
| z | Reh1 | 432 | - | 375-432 | 375-432 | 375-432 | 375-432 | 379-431 |
