# Supplementary material for: Mechanism of completion of peptidyltransferase centre assembly in eukaryotes
Source: eLife. 2019 May 22;8:e44904. doi: 10.7554/eLife.44904 (PMC6579518; doi:10.7554/eLife.44904)
Supplement: Supplementary file 3. [file elife-44904-supp3.docx]

**Supplementary file 3A. Plasmids**

| **Plasmid** | **Description** | **Source** |
| --- | --- | --- |
| pRS316 | *URA3 CEN* |  |
| pRS313 | *HIS3 CEN* |  |
| NEU 023 | uL16 (*URA3 CEN*) | This study |
| NEU 072 | uL16-R98S (*URA3 CEN*) | This study |
| NEU 048 | Nmd3 (*HIS3 CEN*) | This study |
| NEU 055 | Nmd3-Q176A (*HIS3 CEN*) | This study |
| NEU 057 | *NMD3-N269A HIS3 CEN* | This study |
| NEU 067 | *NMD3-N205I HIS3 CEN* | This study |
| NEU 068 | *NMD3-N205D HIS3 CEN* | This study |

**Supplementary file 3B. Primers**

| **Name** | **Sequence** |
| --- | --- |
| RPL10-F | CGGCCGCTCTAGAACTAGTGTCAGGTCCAAGTCTCGTGTT |
| RPL10-R | TATCGATAAGCTTGATATCGAGACTTCTCAGGCCATTGGA |
| RPL10 R98S F | TTGAGTATCAACAAGATGTT |
| RPL10 R98S R | TTGTTGATACTCAAGACATGGAAAGGA |
| NMD3 F | CGGCCGCTCTAGAACTAGTGAACCAAGGCGAAGAGGGAAT |
| NMD3 R | TATCGATAAGCTTGATATCGTCCATCTTCCCAGCATTCCA |
| NMD3 N269A F | TGGGTGCGATTTCTCAATTTGTTCTAT |
| NMD3 N269A R | AAATCGCACCCATAGATTTAGCCAG |
| NMD3 N205I F | CGCAAAAAATCCACGCAGTTAAGATG |
| NMD3 N205I R | CGTGGATTTTTTGCGCATAGAAGAAAT |
| NMD3 N205D F | CGCAAAAAGACCACGCAGTTAAGATGATT |
| NMD3 N205D R | CGTGGTCTTTTTGCGCATAGATCTTTAGG |
| NMD3 Q176A F | TGGAAGCACTGATTTTGAAACATAAT |
| NMD3 Q176A R | CAGTGCTTCCAAAAACAAAAATGTTC |

**Supplementary file 3C. Yeast strains**

| **Name** | **Genotype** | **Source** |
| --- | --- | --- |
| LSG1-TAP | *MAT*α*, ura3, trp1, his3, leu2, YGL099w-TAP (TRP1)* | E. Hurt (Nissan et al., 2002) |
| LW1 | *MAT***a**, *NatMX4::GAL1-RPL10*, *leu2-3*, *leu2-112*, *trp1-1*, *can1-100*, *ura3-1*, *ade2-1*, *his3-11*, *his3-15*, *GAL-RPL10* | This study |
| NE0206 | *MAT***a**, *NatMX4::GAL-RPL10*, *leu2-3*, *leu2-112*, *trp1-1*, *can1-100*, *ura3-1*, *ade2-1*, *his3-11*, *his3-15*, with *uL16-R98S* (*URA3*) | This study |
| NE0209 | *MAT***a**, *NatMX4::GAL-RPL10*, *leu2-3*, *leu2-112*, *trp1-1*, *can1-100*, *ura3-1*, *ade2-1*, *his3-11*, *his3-15*, with *uL16-R98S* (*URA3*), *pRS313* (*HIS3*) | This study |
| NE0211 | *MAT***a**, *NatMX4::GAL-RPL10*, *leu2-3*, *leu2-112*, *trp1-1*, *can1-100*, *ura3-1*, *ade2-1*, *his3-11*, *his3-15*, with *uL16-R98S* (*URA3*), pNmd3 (*HIS3*) | This study |
| NE0233 | *MAT***a**, *NatMX4::GAL-RPL10*, *leu2-3*, *leu2-112*, *trp1-1*, *can1-100*, *ura3-1*, *ade2-1*, *his3-11*, *his3-15*, with *uL16-R98S* (*URA3*), pNmd3-N269A (*HIS3*) | This study |
| NE0235 | *MAT***a**, *NatMX4::GAL-RPL10*, *leu2-3*, *leu2-112*, *trp1-1*, *can1-100*, *ura3-1*, *ade2-1*, *his3-11*, *his3-15*, with *uL16-R98S* (*URA3*)*,* pNmd3-N205I (*HIS3*) | This study |
| NE0237 | *MAT***a**, *NatMX4::GAL-RPL10*, *leu2-3*, *leu2-112*, *trp1-1*, *can1-100*, *ura3-1*, *ade2-1*, *his3-11*, *his3-15*, with *uL16-R98S* (*URA3*)*,* pNmd3-N205D (*HIS3*) | This study |
| NE0239 | *MAT***a**, *NatMX4::GAL-RPL10*, *leu2-3*, *leu2-112*, *trp1-1*, *can1-100*, *ura3-1*, *ade2-1*, *his3-11*, *his3-15*, with *uL16-R98S* (*URA3*)*,* pNmd3-Q176A (*HIS3*) | This study |
